# Supplementary material for: 4-Phenylcoumarin (4-PC) Glucoside from Exostema caribaeum as Corrosion Inhibitor in 3% NaCl Saturated with CO2 in AISI 1018 Steel: Experimental and Theoretical Study
Source: Int J Mol Sci. 2022 Mar 15;23(6):3130. doi: 10.3390/ijms23063130 (PMC8948901; doi:10.3390/ijms23063130)
Supplement: Supplementary file 1 [file ijms-23-03130-s001.zip › ijms-1593453-supplementary.pdf]

Supplementary material

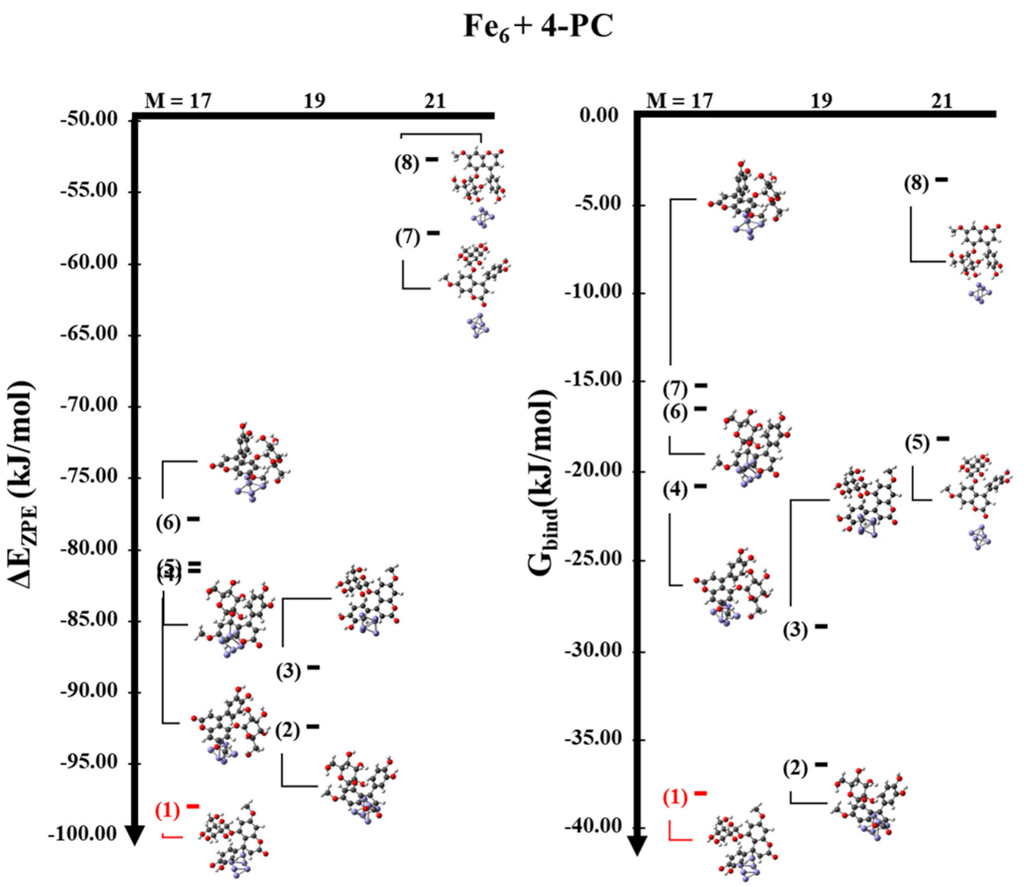

Figure S1. Optimized structures of 4-PC corrosion inhibitor molecule interacting with the Fe_6 cluster at the BPW91-D2/6–311 ++ G (2d,2p) level as function of the ZPE-corrected relative energy ΔE_{ZPE} and G_{bind}.
